# 2021 SNMMI Highlights Lecture: Neuroscience

**Published:** 2021-12

**Authors:** Julie Price

**Affiliations:** Harvard Medical School, and Director, PET Pharmacokinetic Modeling, Athinoula A. Martinos Center for Biomedical Imaging, Massachusetts General Hospital, Boston, MA

## Abstract

*From the Newsline Editor: The Highlights Lecture, presented at the closing session of each SNMMI Annual Meeting, was originated and presented for more than 30 years by Henry N. Wagner, Jr., MD. Beginning in 2010, the duties of summarizing selected significant presentations at the meeting were divided annually among 4 distinguished nuclear and molecular medicine subject matter experts. Each year Newsline publishes these lectures and selected images. The 2021 Highlights Lectures were delivered on June 15 as part of the SNMMI Virtual Annual Meeting. In this issue we feature the lecture by Julie Price, PhD, a professor of radiology at the Harvard Medical School and director of PET Pharmacokinetic Modeling in the Athinoula A. Martinos Center for Biomedical Imaging at the Massachusetts General Hospital (Boston, MA), who spoke on neuroscience highlights from the meeting. Note that in the following presentation summary, numerals in brackets represent abstract numbers as published in* The Journal of Nuclear Medicine *(2021;62[suppl 1]).*

It is my pleasure to present the 2021 SNMMI Neurosciences Highlights lecture. We will start things off with the Kuhl–Lassen award, given annually by the Brain Imaging Council for the past 25 years. The first recipient was Louis Sokoloff, MD, the functional imaging pioneer, who received the award in 1996. The list of awardees over the years constitutes a truly impressive group of scientists and physician scientists. I am immensely humbled to have been this year's recipient and thank the Brain Imaging Council for selecting me for this tremendous honor. The topic of my Kuhl–Lassen lecture was "PET methodology in amyloid imaging."

I want to congratulate the individuals who were selected to take part in the 2021 Neurosciences Young Investigator Award session at this meeting. This is a challenging competition each year, with excellent presentations from our future research leaders. This year the first-place awardee was Emma M. Coomans, the second-place awardee was Matthew Zammit, PhD, and the third-place awardee was Ganna Blazhenets, PhD. Each of their presentations will be featured in this Highlights lecture. I strongly encourage you to view all the Young Investigator Award presentations on the SNMMI Annual Meeting virtual site.

Three oral sessions (Novel Radiotracers and Multimodal Imaging of the Brain, Neurosciences Young Investigator Award Session, and Advances in Clinical Neuroimaging) focused on the neurosciences at this meeting, as did 3 poster sessions covering the basic neurosciences, general neuroscience, and neurology/psychiatry. A rough assessment of these presentations indicated that the topic distribution was largely focused again this year on neurodegeneration and related targets (36%), then on synaptic function and metabolism (23%) and receptor/transporter imaging (14%), with fewer abstracts on inflammation (9%) and brain tumor assessment (4%). The talks highlighted in this lecture will reflect this general distribution.

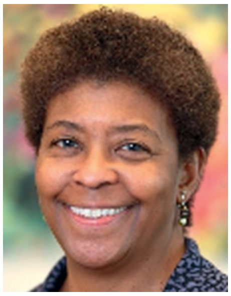

Julie Price, PhD

## Neurotransmitters, Synaptic Function, and Designer Receptors

We will begin with glutamate, the most abundant excitatory neurotransmitter in the vertebrate nervous system. The GluN2B subunit of the *N*-methyl-D-aspartate (NMDA) receptor complex is a therapeutic target for a range of neuropsychiatric disorders, including dementia and schizophrenia. Ongoing efforts to develop GluN2B-specific radiotracers for clinical use have produced several promising compounds. Smart (a Young Investigator) et al. from Yale University PET Center (New Haven, CT), Institute of Pharmaceutical Sciences ETH Zürich (Switzerland), Union Hospital/Tongji Medical College/Huazhong University (Wuhan, China), Jiangsu Institute of Nuclear Medicine (China), and the National Institute of Mental Health (Bethesda, MD) reported on "In vivo comparison of 3 novel radiotracers for the NMDA receptor GluN2B subunit in nonhuman primates" [46]. They compared in rhesus macaques the imaging properties of 3 candidate radiotracers for the GluN2B site: *(R)*-$^{18}$F-OF-Me-NB1, *(R)*-$^{11}$C-NR2B-Me, and *(S)*-$^{18}$F-OF-NB1. In baseline imaging and in blocking scans with selective antagonists for GluN2B and σ1, the researchers compared tracer metabolism in plasma, plasma binding, tissue kinetics, modeling, and selectivity among the 3 compounds. Figure 1 shows the brain distribution for each radiotracer at baseline, as well as the reduction in signal achieved with the blocking scans. *(R)*-$^{11}$C-NR2B-Me and *(S)*-$^{18}$F-OF-NB1 showed similar distribution in brain as well as cortical nondisplaceable binding potential values of 2–3, making them particularly promising candidates for evaluation in humans. Pretreatment with the FTC-146 σ1 antagonist reduced uptake and volume of distribution values in all tracers, most notably for the NR2B ligand, suggesting possible interaction of radiotracers and/or GluN2B targets with σ1 receptors. Additional pharmacologic studies will explore these in vivo properties and interactions.

Afshar et al. from Massachusetts General Hospital/Harvard Medical School (Boston, MA) reported on "Longitudinal assessment of the glutamatergic neurosystem in a fragile X

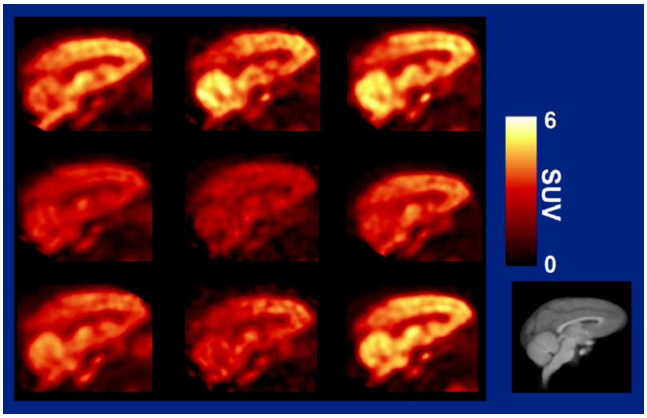

**FIGURE 1.** Three novel radiotracers for the NMDA receptor GluN2B subunit in nonhuman primates. Brain distribution of (left to right) *(R)*-$^{18}$F-OF-Me-NB1, *(R)*-$^{11}$C-NR2B-Me, and *(S)*-$^{18}$F-OF-NB1 PET at (top to bottom) baseline, after GluN2B blocking, and after σ1 blocking. *(R)*-$^{11}$C-NR2B-Me and *(S)*-$^{18}$F-OF-NB1 showed similar distribution in brain as well as cortical binding potential values of 2–3 and are particularly promising candidates for evaluation in humans. Pretreatment with the σ1 antagonist reduced uptake and volume of distribution values of all tracers, most notably for the NR2B ligand, suggesting possible interaction of radiotracers and/or GluN2B targets with σ1 receptors.

knockout mouse model" [118]. Fragile X syndrome is a developmental disorder characterized by learning disabilities and cognitive impairments, caused by mutations of the FMR1 gene. In this study, a cohort of fragile X syndrome mice and age- and sex-matched healthy control mice underwent PET using the allosteric modulator radiotracer $^{18}$F-FPEB to examine mGluR5 expression as well as Morris water maze testing of spatial learning and memory at 4 time points, beginning 34–41 days after birth and extending to about 1 year. The images in Figure 2 show the cumulative distribution of $^{18}$F-FPEB binding to be greater in control mice than in male fragile X knockout mice in cortical and subcortical areas. This was consistent with data that showed a higher binding potential in controls than in knockout mice. Age- and sex-dependent binding potential variations were also observed in different brain areas in the fragile X knockout mice. In male mice, mGluR5 binding potential increased significantly from early adolescence to late adolescence but then decreased in adulthood, suggesting that optimal treatment age may be in adolescence. Increased spatial learning rates during aging was evidenced in the fragile X syndrome mice in mean swim latencies across trials in the Morris water maze studies. The results of these studies provide potential mechanistic insights for design of therapeutic interventions for fragile X and are consistent with the clinical observation that females often have milder symptoms than males. The authors concluded that these findings "reflect the critical brain areas known to be impacted by the progression of the fragile X syndrome, namely striatum, cortex, and hippocampus." In addition, they noted that "biobehavioral vulnerability was predicted on the basis of disease progression and holds exciting potential" as a target for pharmacologic interventions.

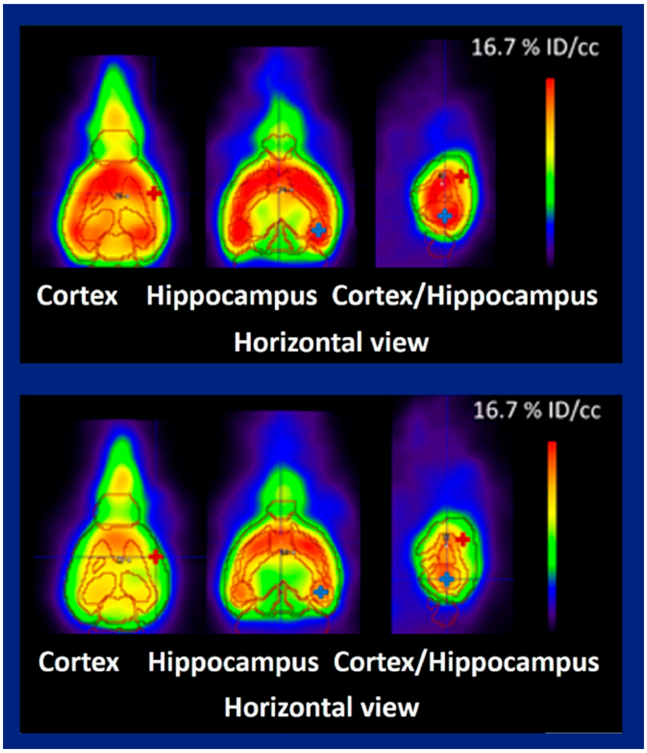

**FIGURE 2.** Longitudinal assessment of the glutamatergic neurosystem in a fragile X knockout mouse model. Cumulative distribution of $^{18}$F-FPEB binding 20–45 min after administration is shown in control male mice (top block) and fragile X syndrome mice (bottom row) in (left to right) cortex, hippocampus, and cortex/hippocampus horizontal views. Differences in distribution reflect the critical brain areas (striatum, cortex, and hippocampus) known to be impacted by progression of fragile X syndrome. Additional biobehavioral studies correlated disease progression as assessed by imaging with symptomatic behaviors and offered potential for identification of targets for pharmacologic intervention.

Gravel et al. from Yale University (New Haven, CT) and F. Hoffmann-La Roche Ltd. (Basel, Switzerland) reported on "Kinetic modeling of novel radiotracers for the GABA transporter-1 (GAT-1) in nonhuman primates" [117]. γ-aminobutyric acid (GABA) is the chief inhibitory neurotransmitter in humans, and GAT-1 is of particular interest because of its potential role in neuropsychiatric disorders. Two novel radiotracers, $^{18}$F-GATT-34 and $^{18}$F-GATT-44, were evaluated at baseline and after blocking with tiagabine, an antiepileptic compound. The uptake images (Fig. 3) for these 2 compounds show that tiagabine blocking decreased the signal for both radiotracers, with moderate success for GATT-34 (48% blocking of specific binding) and better success for GATT-44 (57%–66% blocking of specific binding). The nondisplaceable volume of distribution and average nondisplaceable binding potential values were respectively 0.98 and 0.71 mL/cm$^3$ for GATT-34 and 0.85 and 2.20 mL/cm$^3$ for GATT-44. Injection properties were similar for the 2 tracers. The authors plan to evaluate additional ligands and progress the best to humans.

Yan et al. from the National Institute of Mental Health (Bethesda, MD) and the National Institutes for Quantum and

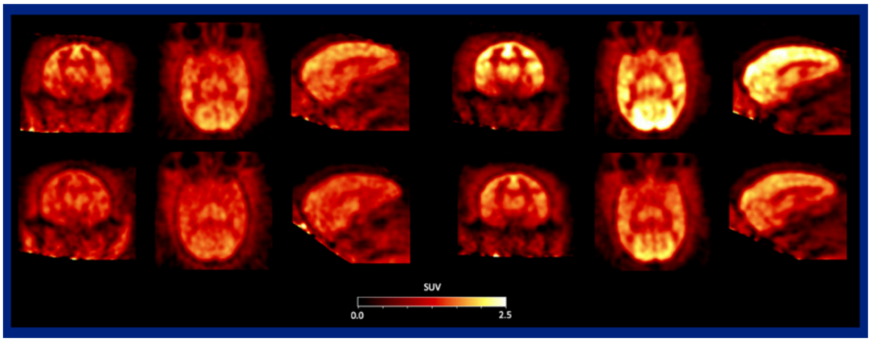

**FIGURE 3.** Kinetic modeling of novel radiotracers for GABA transporter-1 in nonhuman primates. SUV images with $^{18}$F-GATT-34 (left block) and $^{18}$F-GATT-44 (right block) at baseline (top row) and after administration of tiagabine as a blocking agent (bottom row).

Radiological Science and Technology (Chiba, Japan) reported that "$^{11}$C-deschloroclozapine ($^{11}$C-DCZ) is an improved PET radioligand for quantifying a human muscarinic DREADD (Designer Receptors Exclusively Activated by Designer Drugs) expressed in monkey brain" [120]. DREADDs are a powerful technique for selectively manipulating neuronal activity. Recent studies indicate, however, that ligands used for DREADDs, such as clozapine-*N*-oxide or its parent compound clozapine, are not as selective as expected, even at reasonable concentrations. This study was motivated by a previous investigation that found that $^{11}$C-DCZ was superior to $^{11}$C-clozapine ($^{11}$C-CLZ) for imaging DREADDS. The researchers used PET to quantitatively and individually measure signal from transfected receptors, endogenous receptors/targets, and nondisplaceable binding in other brain regions to assess qualities that contribute to the higher signal-to-background ratio of $^{11}$C-DCZ compared to $^{11}$C-CLZ. A genetically modified muscarinic type-4 human receptor was injected into the right amygdala of an 11-year-old male rhesus macaque. $^{11}$C-DCZ and $^{11}$C-CLZ PET scans were conducted at baseline and after receptor blockade, and uptake was quantified relative to the concentration of parent radioligand in arterial plasma at both timepoints. Both tracers had high-affinity displaceable binding to DREADDs relative to endogenous receptor(s) (Fig. 4). The overall signal was 5 times lower for $^{11}$C-DCZ than for $^{11}$C-CLZ, but the background was 10 times lower for $^{11}$C-DCZ, yielding an overall 2-fold gain in signal-to-background ratio for $^{11}$C-DCZ. This higher signal-to-background ratio suggested superior imaging characteristics for $^{11}$C-DCZ, although the percentage of off-target binding (~16%) was similar for the 2 tracers. This is an important study evaluating DREADD in vivo, with implications for behavioral and translational investigations.

## Clinical Neuroimaging: Oncology and COVID-19

This category includes 2 studies aimed at improving therapy response in patients with glioblastoma and a third on COVID-19–related alterations in $^{18}$F-FDG metabolism.

Gan et al. from the Olivia Newton-John Cancer Research Institute/Austin Hospital (Melbourne, Australia) and Royal Brisbane and Women's Hospital (Brisbane, Australia), QIMR Berghofer Medical Research Institute (Brisbane, Australia), and Humanigen, Inc. (Burlingame, CA) reported on "Phase I safety and bioimaging trial of ifabotuzumab in patients with glioblastoma" [104]. Ifabotuzumab is an immunoglobulin G1κ humaneered antibody that targets the EphA3 receptor, which is a tumor-restricted antigen expressed in stroma and in the tumor vasculature of 100% of glioblastoma multiforme. This study is interesting, because it includes evaluation of the biodistribution and pharmacokinetics of $^{89}$Zr-labeled ifabotuzumab, the frequency of EphA3-positive glioblastoma multiforme (tumor targeting), and response rates. Twelve patients (7 men, 5 women; mean age, 51.6 ± 14.24 years) were enrolled, with 6 individuals in each dose cohort (3.5 and 5.25 mg/kg). Treatment was well tolerated with no dose-limiting toxicities observed. Posttreatment MR imaging showed T2/FLAIR changes consistent with treatment effect on tumor vasculature. Figure 5 shows consistent targeting of the tumor microenvironment. The absence of normal tissue uptake of the tracer indicated ideal characteristics for a range of therapeutic

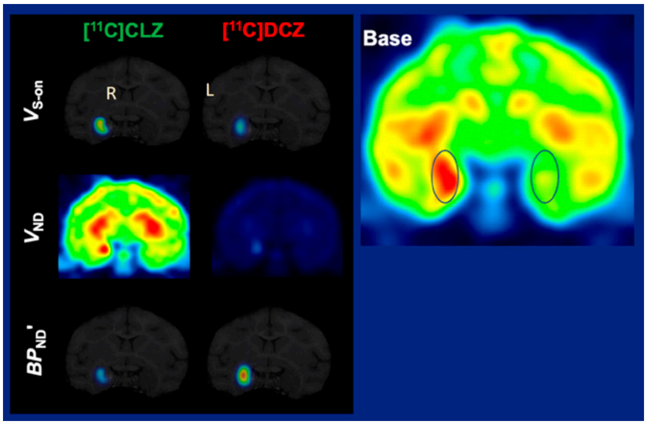

**FIGURE 4.** $^{11}$C-deschloroclozapine ($^{11}$C-DCZ) is an improved PET radioligand for quantifying a human muscarinic Designer Receptors Exclusively Activated by Designer Drug (DREADD) expressed in monkey brain. Left: The signal-to-background ratio of $^{11}$C-DCZ (right column) on PET imaging was found to be ~2-fold higher than that of $^{11}$C-clozapine ($^{11}$C-CLZ) (left column). RIGHT: Baseline $^{11}$C-CLZ image for which right amygdala uptake (circle, left) reflects nonspecific, off-target, and on-target signals, whereas left amygdala (circle, right) reflects only nonspecific and off-target signals. The difference between the uptake in right and left amygdala yields the on-target signal, and the difference between baseline and blocking uptake in other regions yields the off-target signal. In vivo exploration of DREADDs holds broad potential for behavioral and translational investigations.

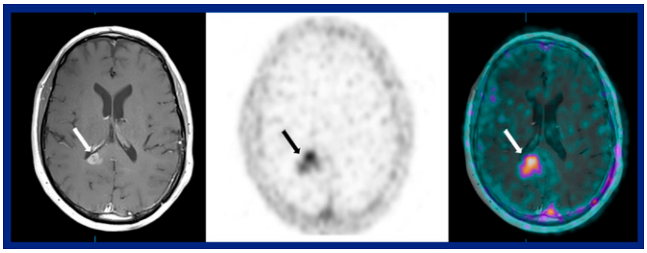

**FIGURE 5.** $^{89}$Zr-ifabotuzumab targeting of tumor microenvironment in glioblastoma multiforme. Left: Post-ifabotuzumab-treatment MR imaging showed T2/FLAIR changes consistent with treatment effect on tumor vasculature (arrow). Middle: $^{89}$Zr-ifabotuzumab showed high specific targeting of the tumor microenvironment in all patients, with no normal tissue uptake, indicating ideal characteristics for a range of therapeutic approaches. Right: PET/MR image. The study evaluated not only the biodistribution and pharmacokinetics of the $^{89}$Zr-labeled immunoglobulin G1κ humaneered antibody, but also the frequency of EphA3-positive glioblastoma multiforme (tumor targeting) and response rates to treatment.

approaches. Median overall survival was 7.17 months (range, 2–28 months), with 3 patients in survival follow-up at the time of the report. Although no objective therapeutic responses were observed (the best being stable disease for 23 weeks), additional studies are planned to evaluate the monoclonal antibody as part of an antibody–drug conjugate in various solid tumor types.

Beinat and Molecular Imaging Program colleagues from Stanford University/Stanford University School of Medicine (CA) reported on "Initial clinical evaluation of $^{18}$F-DASA-23, a PET imaging tracer for evaluation of aberrantly expressed pyruvate kinase M2 (PKM2) in glioblastoma" [99]. PKM2 catalyzes the final step in glycolysis and is highly expressed in glioblastoma cells, with minimal expression in healthy brain. In healthy volunteers, dynamic $^{18}$F-DASA-23 scans exhibited high initial uptake (SUV ~5) in most brain structures, followed by washout over the 60-minute acquisition period. In patients, $^{18}$F-DASA-23 delineated primary brain tumors with a trend toward increasing uptake with increasing tumor grade. This is the first report of the evaluation of a PKM2-specific radiopharmaceutical in humans (Fig. 6). Figure 7 is an example of imaging acquired in an individual in whom $^{18}$F-DASA-23 identified metabolic nonresponse to temozolomide chemotherapy and

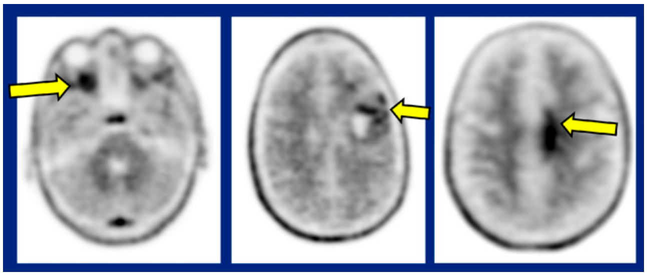

**FIGURE 6.** Initial clinical studies of $^{18}$F-DASA-23 PET for evaluation of aberrantly expressed pyruvate kinase M2 in glioblastoma. $^{18}$F-DASA-23 delineated primary brain tumors with a trend toward increasing uptake with increasing tumor grade. Left: low-grade optical nerve glioma (tissue-to-background ratio maximum [$TBR_{max}$], 2.1). Middle: grade 3 oligodendroglioma ($TBR_{max}$, 2.5). Right: grade 4 glioblastoma ($TBR_{max}$, 2.8).

Avastin antiangiogenic therapy within 1 week of treatment initiation, almost 3 months earlier than contrast-enhanced MR imaging. A promising potential application of $^{18}$F-DASA-23 is in detection of early glycolytic response to therapy in patients with glioblastoma multiforme, with the need for further evaluation of utility. I would like to take a moment to remember a truly exceptional colleague and leader at Stanford University, Sanjiv Sam Gambhir, MD, PhD, who will forever be missed.

Blazhenets et al. from the University of Freiburg (Germany) reported on "Altered regional cerebral function and its association with cognitive impairment in COVID-19: A prospective FDG PET study" [41]. The study included 29 adult patients from the Freiburg Neuro-COVID Registry, with an inclusion criterion of 1 new neurologic symptom related to COVID-19 (disturbed gustation, 100%; disturbed olfaction, 86%; and cranial nerve palsies, 10%). Those with premorbid neurologic conditions, such as dementia, were excluded. Participants underwent neurologic examination, a cognitive test battery, cerebrospinal fluid investigation, and multimodal imaging. Fifteen of the 29 patients with a least 2 new neurologic symptoms underwent cerebral $^{18}$F-FDG PET imaging. Visual reads of the resulting images indicated that 10 of the 15 showed pathologic results. The investigators compared these subjects with a group of controls using principal component analysis, which yielded a distinctive COVID-19–related FDG spatial covariance pattern involving multiple brain

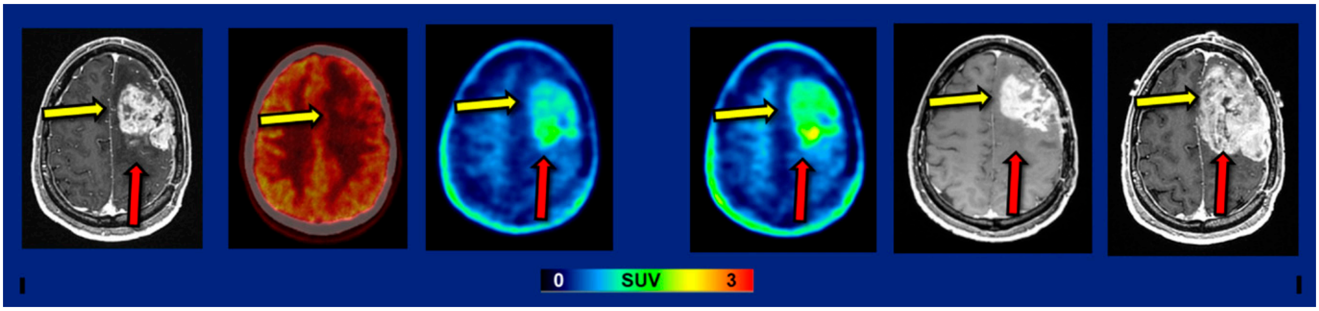

**FIGURE 7.** $^{18}$F-DASA-23 PET in early evaluation of glioblastoma. $^{18}$F-DASA-23 identified a metabolic nonresponder to temozolomide chemotherapy and Avastin antiangiogenic therapy within 1 week of treatment initiation, almost 3 months earlier than contrast-enhanced MR imaging. Left to right: $T_1$ + C MR imaging at 15 days pretreatment, $^{18}$F-FDG PET at 12 days pretreatment, $^{18}$F-DASA-23 PET at 3 days pretreatment, $^{18}$F-DASA-23 PET at 6 days posttreatment, $T_1$ + C MR at 6 days posttreatment, and $T_1$ + C MR at 90 days posttreatment.

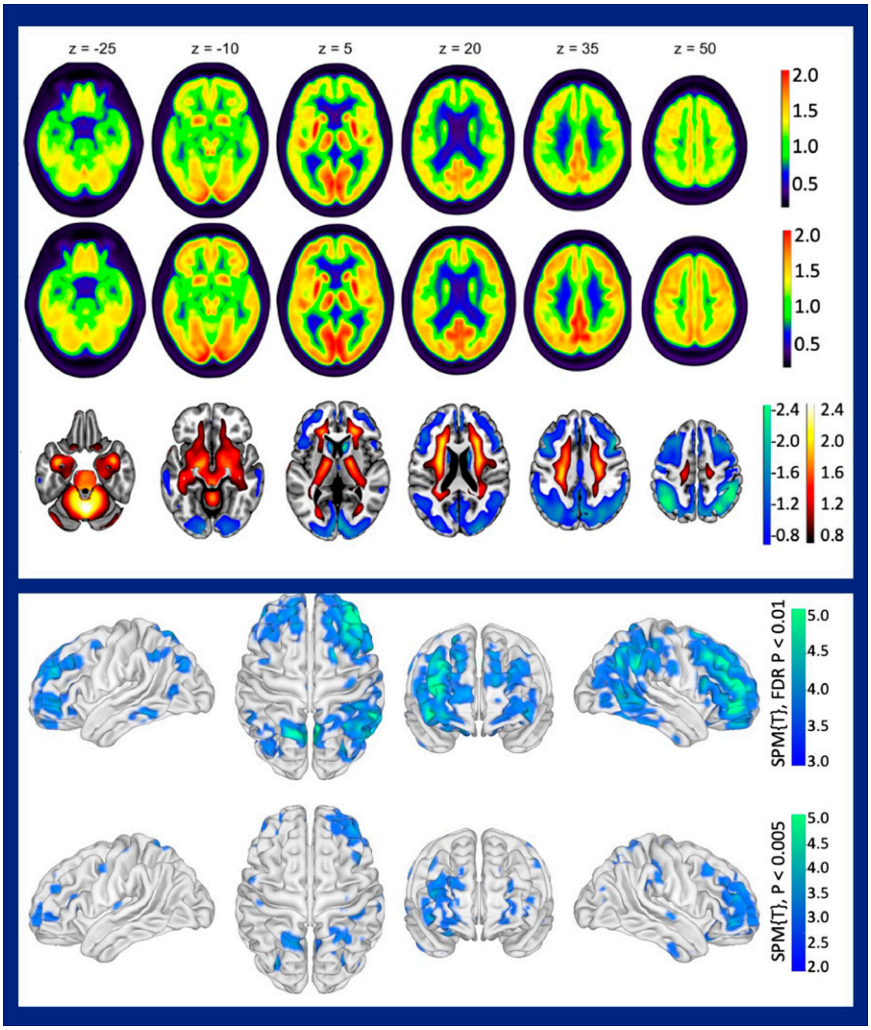

**FIGURE 8.** $^{18}$F-FDG PET and altered regional cerebral function and cognitive impairment in patients after COVID-19. Top block: $^{18}$F-FDG PET in COVID patients (top row) and a control group (middle row). COVID-19–related spatial covariance patterns engaged multiple brain regions and were characterized by negative weights (hypometabolism) in widespread neocortical areas (bottom row). Bottom block: Statistical parametric mapping in subacute COVID patients (top row) and at 6-month follow-up (bottom row), with higher pattern expression associated with worse cognitive performance. Although significant recovery of regional neuronal function and cognition was evident, COVID-19–related residuals were still measurable even 6 months after recovery.

regions and characterized by negative weights (hypometabolism) in widespread neocortical areas (positive weights = preserved regions), brain stem, cerebellum, white matter, and mesiotemporal structures and negative weights in widespread neocortical areas (with frontoparietal predominance) (Fig. 8). Conventional statistical parametric mapping analysis also showed widespread frontoparietal-dominant neocortical hypometabolism, whereas there were no hypermetabolic clusters. In the 8 COVID-19 patients presented for follow-up, the mean pattern expression score at the chronic stage was significantly lower than in the subacute stage. Although significant recovery of regional neuronal function and cognition was clearly evident in these individuals, COVID-19–related residuals were still measurable even 6 months after recovery. Higher $^{18}$F-FDG pattern expression was also associated with worse cognitive performance (i.e., COVID-19–related pattern expression score versus Montreal Cognitive Assessment global cognitive score, adjusted for education). This is a very important study, as it quantifies the neurologic impact of the COVID-19 infection over time. The authors concluded that "post–COVID-19 patients with persistent cognitive complaints should be presented to a neurologist and possibly allocated to cognitive rehabilitation programs." Images from this presentation were named as the winner of the prestigious SNMMI Image of the Year award for 2021.

## Neurodegeneration

Coomans et al. from Vrije Universiteit Amsterdam/Amsterdam UMC (The Netherlands), Lund University (Sweden), Rodin Therapeutics, Inc. (Boston, MA), and University College London Institutes of Neurology and Healthcare Engineering (UK) reported that "In vivo tau pathology is associated with synaptic loss and altered synaptic function" [43]. The authors applied a novel multimodality approach in amyloid-positive Alzheimer disease subjects from the Amsterdam Dementia Cohort who underwent dynamic 130-minute $^{18}$F-flortaucipir PET, dynamic 60-minute $^{11}$C-UCB-J PET with arterial sampling, and 2 × 5-minute resting-state MEG measurements. Figure 9 demonstrates spatial overlap and differences between tau pathology and synaptic loss observed respectively by T1 MR imaging, $^{18}$F-flortaucipir PET, and $^{11}$C-UCB-J PET. The locations of increases in distribution of the PET ligands were markedly different. Across subjects, higher regional $^{18}$F-flortaucipir uptake was associated with lower $^{11}$C-UCB-J uptake, consistent with lower synaptic density. Within subjects, the association between $^{18}$F-flortaucipir and $^{11}$C-UCB-J ligand binding was dependent on the within-subject neocortical tau load or degree of neurodegeneration. Both higher $^{18}$F-flortaucipir and lower $^{11}$C-UCB-J uptake were associated with altered synaptic function, indicative of slowing of oscillatory activity, most pronounced in the occipital lobe. These results indicated that in Alzheimer disease tau pathology is closely associated with reduced synaptic density and synaptic dysfunction. This study illuminates ways in which $^{11}$C-UCB-J synaptic parameters relate to other imaging parameters in neurodegeneration and

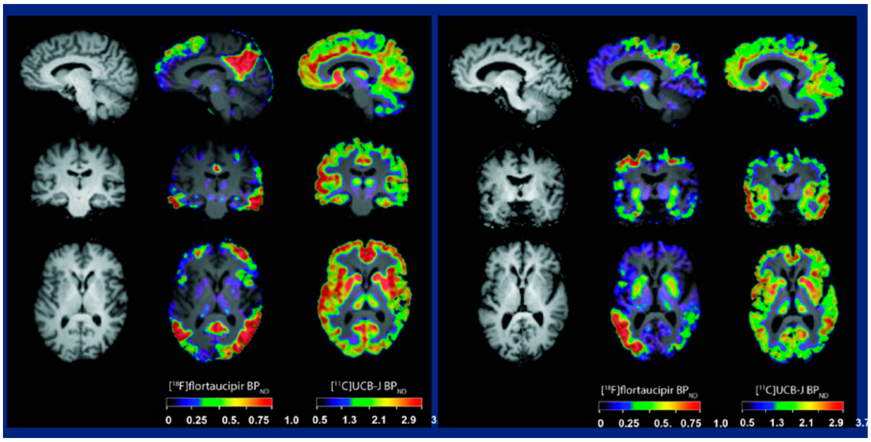

**FIGURE 9.** In vivo imaging of tau pathology associated with synaptic loss and altered synaptic function. Images demonstrate spatial overlap between tau pathology and synaptic loss observed in T1 MR imaging (left column in each block) and binding potential maps of $^{18}$F-flortaucipir (middle column) and $^{11}$C-UCB-J PET/CT (right column). Left block acquired in a 50–55-year-old woman with a Mini Mental State Examination (MMSE) score of 22. Right block acquired in a 55–60-year-old man with an MMSE = 24. Both higher $^{18}$F-flortaucipir and lower $^{11}$C-UCB-J uptake were associated with altered synaptic function, indicative of slowing of oscillatory activity, most pronounced in the occipita l lobe. These results indicated that tau pathology in Alzheimer disease is closely associated with reduced synaptic density and synaptic dysfunction.

continues to build on information that can inform future therapeutic strategies.

Zammit et al. from the University of Wisconsin Madison, University of Pittsburgh (PA), University of Cambridge (UK), and Washington University in St. Louis (MO) reported that "Neurofibrillary tau emerges in adults with Down syndrome during the earliest stages of Aβ accumulation" [42]. Adults with Down syndrome are predisposed to early Alzheimer disease, accumulating amyloid-β early in life. The aim of the study was to evaluate neurofibrillary tau deposition (using $^{18}$F-AV-1451 or $^{18}$F-flortaucipir PET) in adults with Down syndrome classified as amyloid-negative (A–) or amyloid-positive (A+) at subthreshold levels. Figure 10 is an SUV ratio ($SUV_r$) difference image between the subthreshold A+ and A– participants, with greater uptake difference indicating elevated AV-1451 retention in the subthreshold A+ group. Even at very low amyloid load levels, the subthreshold A+ group exhibited significantly higher tracer uptake in Braak regions I–III. Higher off-target binding in choroid plexus and basal ganglia was observed in the subthreshold A+ group compared to the whole sample of A–, whereas the $SUV_r$ difference in off-target binding was lower in the age-matched group, indicating age-related AV-1451 off-target binding in Down syndrome. These findings indicate that neurofibrillary tau burden is evident even in the early Braak stage regions during the subthreshold amyloid-β accumulation phase in Down syndrome, suggesting a short latency between the onset of amyloid-β and the spread of neurofibrillary tau.

High neocortical tau is rarely observed in the absence of high amyloid-β in Alzheimer disease. We also know that there is believed to be a threshold of amyloid beyond which tau accumulation progresses with cognitive decline. Krishnadas et al. from Austin Hospital Heidelberg, the University of Melbourne Parkville, Florey Institute of Neurosciences and Mental Health Parkville, CSIRO Biomedical Imaging Health and Biosecurity Flagship Parkville, and the Australian Dementia Network (Victoria; all in Australia) reported on "Discordant low amyloid-β PET and high neocortical tau PET retention" [100]. In a large cohort (466 participants) from the Australian Imaging Biomarkers and Lifestyle study of aging and Alzheimer disease, 287 individuals were found to have low/negative amyloid-β on $^{18}$F-NAV4694 PET (centiloid < 25), 12 of these had both low/negative amyloid-β PET and quantitatively high neocortical $^{18}$F-MK6240 tau,

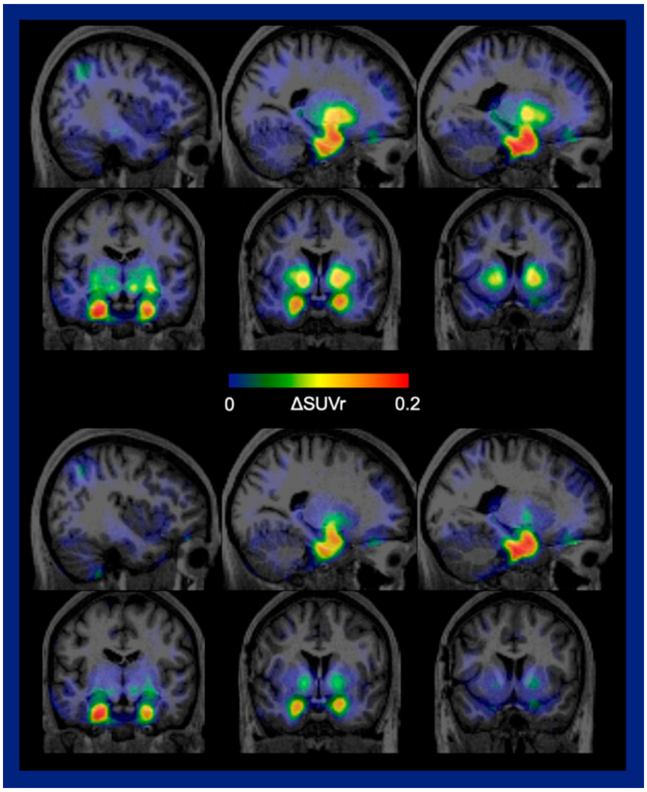

**FIGURE 10.** Neurofibrillary tau in adults with Down syndrome. $^{18}$F-AV-1451 PET $SUV_r$ difference imaging between amyloid-negative and subthreshold amyloid-positive participants (top block, whole sample; bottom block; age-matched sample). Even at very low amyloid load levels, the subthreshold amyloid-positive group exhibited significantly higher tracer uptake in Braak regions I–III, indicating that neurofibrillary tau burden is evident even in the early Braak stage regions during the subthreshold amyloid-β accumulation phase in Down syndrome.

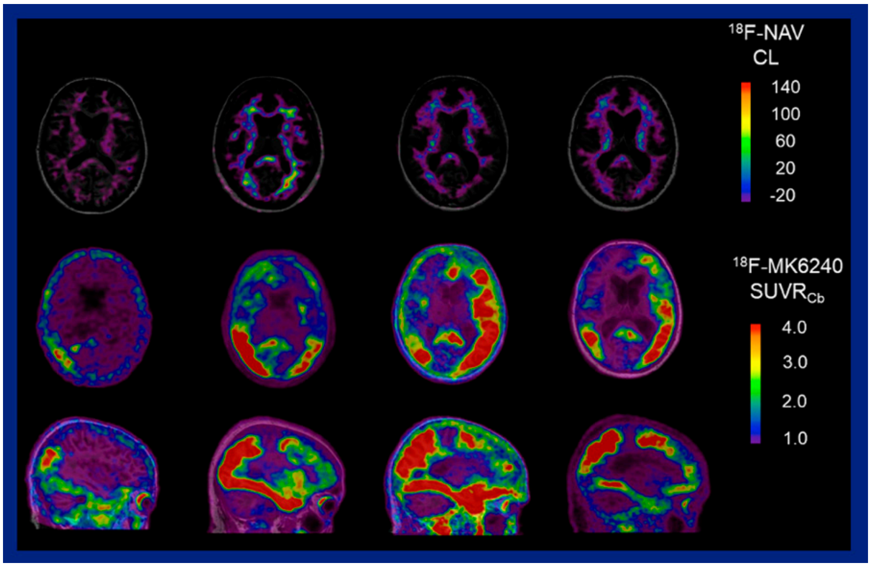

**FIGURE 11.** Discordant low amyloid-β PET and high neocortical tau PET retention. Only 4 out of 466 individuals in a large Alzheimer disease study were found to have low/negative amyloid-β PET and quantitatively high neocortical tau with visually positive tau PET consistent with Alzheimer disease. These individuals (left to right, assessed as clinically unimpaired, with mild cognitive impairment, with mild cognitive impairment, and with Alzheimer disease) were imaged with $^{18}$F-NAV4694 (top row) and $^{18}$F-MK6240 (middle and bottom rows). Despite this atypical presentation, alternative biomarkers were suggestive of Alzheimer disease in all 4 participants.

and only 4 of the 12 had low/negative amyloid-β PET and quantitatively high neocortical tau with visually positive tau PET consistent with Alzheimer disease. Figure 11 shows example PET images in 4 participants with discordant low amyloid-β PET ($^{18}$F-NAV4694) and high neocortical tau ($^{18}$F-MK6240). Despite this atypical presentation, alternative biomarkers were suggestive of Alzheimer disease in all 4 participants. The authors proposed that in these very rare instances of discordance, "the amyloid-β PET ligand may not be detecting Aβ, maybe due to a different conformation of the aggregates." They called for larger studies to validate the findings, as well as genetic testing and postmortem correlation to enhance understanding and determine whether this constitutes a different form of Alzheimer disease.

Previous studies have shown that amyloid-β likely promotes the spread of tau beyond the medial temporal lobe. However, the amyloid-β levels necessary for tau to spread in the neocortex remain unclear. Dore et al. from CSIRO Heidelberg, Austin Hospital Heidelberg, CSIRO Herston, and the Florey Institute of Neuroscience and Mental Health (Melbourne; all in Australia) reported on the "Relationship between amyloid and tau levels and its impact on tau spreading" [116]. In the same large cohort from the Australian Imaging Biomarkers and Lifestyle study, participants underwent tau imaging with $^{18}$F-MK6240 and amyloid-β imaging with $^{18}$F-NAV4694. Amyloid-β scans were quantified on the centiloid scale with a cutoff of 25 for abnormal levels of amyloid-β (A+). The prevalence of abnormal tau levels along the centiloid continuum was also determined. Below 50 on the centiloid scale, the prevalence of individuals with an abnormal tau scan was low in mesial temporal and rare in temporoparietal areas. Above 50 centiloids, the prevalence of abnormal tau levels accelerated in all areas. In cognitively unimpaired individuals, the prevalence of abnormal tau increased along the centiloid continuum. The highest prevalence of tau abnormality was found in the entorhinal cortex, reaching 40% at 40 and 80% at 60 on the centiloid scale. Outside the entorhinal cortex, abnormal levels of cortical tau on PET were rarely found with amyloid-β levels below 40. Moderate amyloid-β levels, then, appear to be required before neocortical tau becomes detectable.

## Conclusion

I would like to end this lecture by thanking my colleagues for this honor and paying tribute to Henry N. Wagner, Jr., MD, for his stewardship, mentorship, and extraordinary efforts to advance the field of nuclear medicine.
